# The risk perception of COVID-19 and vaccine uptake among patients with chronic illnesses at a tertiary health facility in Nigeria

**DOI:** 10.4314/gmj.v58i3.4

**Published:** 2024-09

**Authors:** Mojirola M Fasiku, Oluwatoyosi R Aibinuomo, Oluwatomi Akande, Tolulope G Kayode, Medinat O Aliu-Ayinde, Ige A Adejoro, Maryam A Jimoh, Tanimola M Akande

**Affiliations:** 1 Department of Epidemiology and Community Health, University of Ilorin Teaching Hospital; 2 ESC Clermont, 4 Boulevard Trudaine, Clermont-Ferrand France

**Keywords:** Vaccine-Uptake, Risk-Perception, COVID-19, Chronic-illnesses, Nigeria

## Abstract

**Objectives:**

This study assessed the risk perception of COVID-19 and the uptake of the COVID-19 vaccine among patients with chronic illnesses in a tertiary health facility.

**Design:**

A hospital-based cross-sectional study.

**Setting:**

The outpatient clinics in a tertiary health facility in Ilorin, North-Central Nigeria

**Participants:**

Patients with chronic diseases attending outpatient clinics in UITH, Ilorin from November- December 2022, excluding patients under 18 years of age, using simple random sampling by balloting for outpatient clinics, proportional allocation for participants from each clinic, and systematic sampling method for eligible respondents.

**Main outcome measure:**

Risk perception of COVID-19 and vaccine uptake among patients with chronic illnesses in Nigeria

**Results:**

Respondents believed that older people were most at risk of COVID-19. Over two-thirds, 278 (69.5%) of the respondents had received the COVID-19 vaccine. Fear of the unknown (36.0%) and fear of side effects 30 (24.6%) were the most common reasons for not taking the vaccine. Those married were more likely to have received at least one dose of the vaccine (p=0.007).

**Conclusion:**

COVID-19 risk perception and COVID-19 vaccine uptake were relatively above average. Fear of the unknown and side effects were significant reasons for not taking the vaccines.

**Funding:**

None declared

## Introduction

Coronavirus disease 2019 (COVID-19), stemming from (Severe Acute Respiratory Syndrome Coronavirus 2) SARS-CoV-2, was declared a pandemic by the WHO on March 11, 2020.^1^,^2^ In Kwara State, Nigeria. The first case emerged in April 2020 at the University of Ilorin Teaching Hospital, triggering anxiety and stress. The Nigerian government promptly enforced control measures, including face masks, hand washing, social distancing, lockdowns, and surveillance. Vaccination commenced on March 6, 2021, beginning with AstraZeneca and later including Pfizer and Moderna vaccines.^3^-^8^ Risk perception involves intuitive hazard assessments shaped by biological, social, cultural, geographical, economic, and political factors. Determinants of COVID-19 risk perception include knowledge about the virus and vaccines, personal experience, social amplification, trust in government, science, medical professions, decision-making ability, and political ideology. Understanding perceived risk is vital for effectively controlling outbreaks, especially among those with chronic illnesses, who face higher risk and mortality rates.^9^-^14^

Preventative measures such as sanitiser use, glove and mask-wearing, social distancing, and vaccination are essential. Vaccination is a public health priority, safeguarding patients with chronic illnesses and reducing infection severity, even among the unvaccinated, through herd immunity.^15^-^22^ Reports indicate low COVID-19 vaccine acceptance and uptake among patients with chronic illnesses. A study in Ethiopia's tertiary health facility revealed that only 26.9% of chronic disease patients had received a COVID-19 vaccine dose. Factors affecting uptake included age, education, COVID-19 history, and testing. Reasons for not getting vaccinated included doubts about efficacy, fear of adverse effects, and concerns about the vaccine causing disease.^23^,^24^

About 62.7% of study participants from a study conducted in Ghana were willing to take the COVID-19 vaccine.^25^ It was, however, suggested that future studies be carried out on COVID-19 vaccination in Africa in another study.^26^ Studies carried out in Nigeria show that less than half of individuals with chronic medical conditions in Nigeria showed a positive attitude toward COVID-19 vaccination. Biological and sociopolitical factors influenced hesitancy toward the COVID-19 vaccine. Biological concerns included fears of the vaccine worsening underlying conditions, harmful physiological consequences, insufficient testing, and perceived vaccine ineffectiveness. Sociopolitical issues involved misconceptions about vaccines, such as COVID-19 treatments, mistrust of manufacturers and government, and COVID-19 misinformation. A positive attitude toward vaccination was associated with confidence in the government, education, income, COVID-19 knowledge, and living conditions. To promote a positive vaccine attitude, the government must improve health instructions, demonstrating transparency and effective communication to maintain public trust and confidence.^27^-^30^

There is a lack of studies on COVID-19 vaccine uptake and determinants among patients with chronic illnesses in this area. Understanding the determinants of vaccine uptake in this geographical setting can provide evidence for solutions and inform policies, especially in low-income countries, where those with chronic illnesses are more vulnerable to developing COVID-19.

Therefore, this study assessed the risk perception of COVID-19, vaccine uptake, and its determinants among patients with chronic illnesses who attended Consultants' Outpatient Clinics at the University of Ilorin Teaching Hospital, North Central Nigeria.

## Methods

### Study Area

The University of Ilorin Teaching Hospital (UITH), located in Kwara state, is a federal tertiary hospital catering to patients from Kwara and nearby states. Housing 650 beds, it admits roughly a thousand in-patients monthly. It operates 21 outpatient clinics, excluding pediatric services, on rotating schedules. In 2018, 2019, and 2020, these clinics treated approximately 6152, 7955, and 3322 patients, respectively.^25^

### Study design

The research is a cross-sectional study carried out at UITH, Ilorin, among patients with chronic illnesses attending outpatient clinics from November to December 2022.

### Study Population

Adult patients with chronic diseases attending the UITH's outpatient clinics in Ilorin, excluding those under 18.

### Sample size and technique

The minimum sample size was scientifically determined using Kish Leslie formula with a standard deviation at the 95% confidence interval (1.96) with a p of 44% for calculating sample size for a population of less than 10,000.^31^

Out of the 21 outpatient clinics, 14 were randomly chosen via balloting without replacement. Participants were then selected proportionally from each clinic based on patient attendance. Eligible participants were chosen through systematic sampling, starting with a randomly selected individual and then selecting every nth participant until the required sample size was reached. The sampling interval was determined based on the number of registered patients for each clinic divided by the number of patients to be interviewed in the clinic following proportional allocation.

### Data collection

Data was gathered through a semi-structured questionnaire based on previous literature, utilising the KOBO Collect App. 18,19,24,27,32,34 Four trained Research Assistants conducted the interviews.

### Data Management

Data was manually sorted and computerised for analysis using IBM SPSS version 21. Descriptive and inferential statistics, including Chi-square tests, were performed. Significance was determined at a p-value <0.05 with a 95% confidence interval.

### Inclusion and exclusion Criteria

Patients who had been attending the clinic for at least a year before COVID-19 and who were 18 years and above were included in the study. Patients with debilitating illnesses who could not participate in the study despite meeting the inclusion criteria were exempted from the study.

### Ethical Issues

The Ethical Review Committee of UITH, Ilorin, gave ethical approval before starting the study (ERC PAN/2022/08/0328). Participants provided informed consent after being briefed on the study's purpose. Confidentiality of the information collected was ensured.

## Results

Table 1 revealed that the highest proportion, 117(29.3%), of respondents were those in the age group 56-65 years. The mean age was 59 ± 13.1 years. A greater proportion of respondents were females, 225(56.2%). The majority of the respondents were married, 330(82.5%). Most of the respondents had tertiary education, 205 (51.2%). The majority of the respondents, 284(71.3%), were employed.

**Table 1:** Socio-demographic characteristics of respondents

Socio-demographic Characteristics	Frequency (n=400)	Percentage	
		(n=100%)	
**Age (years)**			
<35	14	3.5%	
35-45	49	12.3%	
46-55	87	21.7%	
56-65	117	29.3%	
66-75	98	24.5%	
>75	35	8.7%	
**Mean age (±SD)**			59 (13.1)
**Minimum age**			20
**Maximum age**			95

**Sex**			
Female	225	56.2%	
Male	175	43.8%	

**Marital Status**			
Single	7	1.8%	
Married	330	82.5%	
Widowed	60	15.0%	
Divorced/Separated	3	0.7%	

**Type of marriage**	**(n=330)**		
Monogamy	251	83.7%	
Polygamy	79	26.3%	

**Religion**			
Christianity	150	37.5%	
Islam	249	62.3%	
Traditional religion	1	0.2%	

**Educational Status**			
None	46	11.5%	
Primary	37	9.3%	
Secondary	112	28.0%	
Tertiary	205	51.2%	

**Employment Status**			
Employed	284	71.3%	
Unemployed	115	28.7%	

**Monthly Income (Naira)**	98	24.5%	
<30000	149	37.3%	
30000-79999	84	21.0%	
80000-129999	30	7.5%	
130000-179999	39	9.7%	
>180000			54,500.0
Median (IQR)			(70,000.0)

**Place of Residence**			
Within Ilorin	346	86.5%	
Outside Ilorin	54	13.5%	

Those with a monthly income of between **>30000** Naira and less than 80000 Naira had the greatest proportion of respondents, 149(37.3%).

Table 2 shows that almost half, 177 (44.3%) of the respondents agreed that they were at risk of being infected with COVID-19 while attending the clinic in UITH. About one-third, 130 (32.5%) agreed they were likely to become sick with the new COVID-19. This is closely followed by those 116 (29.0%) who disagreed that they would likely become sick with the new COVID-19. More than half, 228 (57.0%) and 231(58.8%) of respondents agreed that using a facemask and regular washing of hands with water and soap could prevent infection with COVID-19, respectively. However, 2 (0.5%) strongly disagreed with the idea of regularly washing hands with soap and water as a way of preventing infection with COVID-19. About half, 206 (51.5%) of the respondents agreed that not sitting close to people could prevent COVID-19, and 200 (50.0%) agreed that COVID-19 could be contracted if no preventive measure was taken.

**Table 2:** Risk Perception towards COVID-19

Perception	Strongly Agree n (%)	Agree n (%)	Neutral n (%)	Disagree n (%)	Strongly Disagree n (%)
**At risk of being infected with COVID-19 while attending clinic in UITH**	84 (21.0)	177 (44.3)	61 (15.3)	73 (18.2)	5 (1.2)
**Likely to become sick with the new COVID-19**	48 (12.0)	130 (32.5)	87 (21.8)	116 (29.0)	19 (4.7)
**Use of facemask can prevent infection with COVID-19**	106 (26.5)	228 (57.0)	44 (11.0)	18 (4.5)	4 (1.0)
**Regular washing of hands with water and soap can prevent infection with COVID-19**	128 (32.0)	231 (58.8)	33 (8.2)	6 (1.5)	2 (0.5)
**Not sitting close to people can prevent COVID-19**	105 (26.3)	206 (51.5)	60 (15.0)	22 (5.5)	7 (1.7)
**Can contract COVID-19 if no preventive measure is taken**	93 (23.2)	200 (50.0)	72 (18.0)	29 (7.3)	6 (1.5)

**Figure 1:**
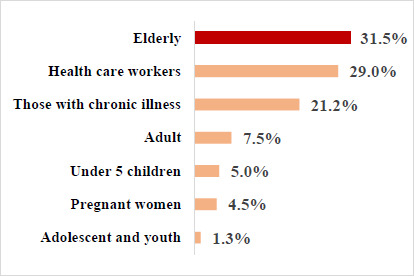
Respondents' view on most at risk of COVID-19

The highest proportion of the respondents (31.5%) believed that older people were the most at risk of COVID-19. While 29.0% and 21% thought that healthcare workers and those with chronic illnesses were most at risk of COVID-19, respectively. (Figure 1)

Table 3 shows that 69(17.2%) respondents had ever taken a COVID-19 test. However, only about 7(10.1%) of those tested were positive for COVID-19. Over two-thirds, 278 (69.5%) of the respondents had received the COVID-19 vaccine. The highest proportion, 135 (48.6%) of those who received the COVID-19 vaccine, had only taken the first dose, while 112 (40.3%) had up to the booster dose. However, of the 122 (30.5%) respondents that had not received the COVID-19 vaccine, fear of the unknown (36.0%) was the most common reason given for not taking the vaccine, closely followed by the fear of side effects 30 (24.6%).

**Table 3:** COVID-19 Status and Vaccine Uptake

Variable	Frequency	Percentage
	(n=400)	(n=100%)
**Ever taken COVID-19 test**		
No	331	82.8%
Yes	69	17.2%

**Ever had a positive result**	***(n=69)***	
No	62	89.9%
Yes	7	10.1%

**Ever been hospitalised for COVID-19**	***(n=7)***	
No	4	57.1%
Yes	3	42.9%

**Ever received COVID-19 vaccine**		
No	122	30.5%
Yes	278	69.5%

**Which dose was received**	***(n=278)***	
1^st^ dose only	135	48.6%
1^st^ and 2^nd^ dose only	31	11.1%
1^st^, 2^nd^ and booster dose	112	40.3%

**Most common reason for not receiving the vaccine**	***(n=122)***	
Fear of side effect	30	24.6%
Fear of the unknown	44	36.0%
Don't need it	25	20.5%
Not safe	4	3.3%
*Others	19	15.6%

Table 4a shows a statistically significant relationship between marital status and COVID-19 vaccine uptake among respondents. Those who were married were more likely to have received at least one dose of the vaccine (*p=0.007*). However, all the other socio-demographic variables had no statistically significant relationship with COVID-19 vaccine uptake among respondents.

**Table 4a:** Factors associated with COVID-19 vaccine uptake among respondents

Socio-demographic	Never taken vaccine (%) (n=122)	Taken at least 1 vaccine dose (%) (n=278)	χ^2^	p-value
**Age**			8.235	0.144
<35	8 (6.6%)	6 (2.2%)		
35-45	17 (13.9%)	32 (11.5%)		
46-55	27 (22.1%)	60 (21.6%)		
56-65	34 (27.9%)	83 (29.9%)		
66-75	23 (18.9%)	75 (26.9%)		
>75	13 (10.6%	22 (7.9%)		

**Sex**			0.127	0.722
Female	67 (54.9%)	158 (56.6%)		
Male	55 (45.1%)	120 (43.2%)		

**Marital Status**			12.242	**0.007**
Single		1 (0.4%)		
Married		233 (83.8%)		
Widowed		43 (15.5%)		
Divorced/Separated		1 (0.4%)		

**Educational Status**			1.368	0.713
None	11 (9.0%)	35 (12.6%)		
Primary	13 (10.7%)	24 (8.6%)		
Secondary	34 (27.9%)	78 (28.1%)		
Tertiary	64 (52.5%)	141 (50.7%)		

**Employment Status**			0.887	0.401
Employed	83 (68.0%)	202 (72.7%)		
Unemployed	39 (32.0%)	76 (27.3%)		

**Number of chronic diseases**			3.825	0.281
	86 (70.5%)	186 (66.9%)		
	28 (23.0%)	83 (29.9%)		
	7 (5.7%)	8 (2.8%)		
	1 (0.8%)	1 (0.4%)		

**Table 4b:** Factors associated with COVID-19 vaccine uptake among respondents

Risk Perception of COVID-19	Never taken vaccine (%) (n=122)	Taken at least 1 vaccine dose (n(%)), (n=278)	χ^2^	p-value
**Believes he/she is at risk of being infected while attending UITH clinic**			**10.762**	**0.029**
Strongly agree	20 (16.4%)	64 (23.0%)		
Agree	46 (37.7%)	131 (47.1%)		
Neutral	25 (20.5%)	36 (12.9%)		
Disagree	30 (24.6%)	43 (15.5%)		
Strongly disagree	1 (0.8%)	4 (1.5%)		

**Believes he/she is more likely to become sick with the new variant**			**5.630**	**0.229**
Strongly agree	12 (9.8%)	36 (12.9%)		
Agree	32 (26.2%)	98 (35.3%)		
Neutral	31 (25.4%)	56 (20.1%)		
Disagree	39 (32.0%)	77 (27.7%)		
Strongly disagree	8 (6.6%)	11 (4.0%)		

**Believes use of facemask can prevent infection**				**0.000**
Strongly agree	15 (12.3%)	91 (32.7%)		
Agree	69 (56.6%)	159 (57.2%)		
Neutral	26 (21.3%)	18 (6.5%)		
Disagree	11 (9.0%)	7 (2.5%)		
Strongly disagree	1 (0.8%)	3 (1.1%)		

**Believes regular hand washing with water and soap can prevent infection**			**30.653**	**0.000**
Strongly agree	19 (15.6%)	109 (39.2%)		
Agree	81 (66.4%)	150 (54.0%)		
Neutral	18 (14.8%)	15 (5.4%)		
Disagree	4 (3.2%)	2 (0.7%)		
Strongly disagree	0 (0.0%)	2 (0.7%)		

**Believes not sitting close to people can prevent infection**			**17.981**	**0.001**
Strongly agree	18 (14.8%)	87 (31.3%)		
Agree	64 (52.5%)	142 (51.1%)		
Neutral	26 (21.3%)	34 (12.2%)		
Disagree	11 (9.0%)	11 (4.0%)		
Strongly disagree	3 (2.5%)	4 (1.4%)		

**Believes he/she can contract the virus if no prevent measure is taken**			**31.188**	**0.000**
Strongly agree	13 (10.6%)	80 (28.8%)		
Agree	60 (49.2%)	140 (50.4%)		
Neutral	28 (23.0%)	44 (15.8%)		
Disagree	19 (15.6%)	10 (3.6%)		
Strongly disagree	2 (1.6%)	4 (1.4%)		

Those who agreed that there is a risk of infection while attending a clinic in UITH, using a face mask to prevent COVID-19, regular hand washing, and not sitting close to people to prevent COVID-19 all had a statistically significant relationship with the uptake of the COVID-19 vaccine (p=0.029, 0.000, 0.000, and 0.001, respectively).

There is no statistically significant relationship between the respondents who believed they were more likely to become sick with the new variant and the uptake of the COVID-19 vaccine.

The number of chronic diseases was statistically significant in association with having positive COVID-19 test results (Table 5). Those with two chronic diseases were more likely to have a positive COVID-19 result (p=0.000)

**Table 5:** Factors associated with having a positive COVID-19 result among respondents

Variable	Never had COVID-19 (n=62)	Had COVID-19 before (%) (n=7)	χ^2^	p-value
**Age**			**6.979**	**0.727**
<35	2 (3.2%)	0 (0%)		
35-45	4 (6.5%)	1 (14.3%)		
46-55	15 (24.2%)	1 (14.3%)		
56-65	25 (40.3%)	1 (14.3%)		
66-75	13 (21.0%)	3 (42.8%)		
>75	3 (4.8%)	1 (14.3%)		

**Sex**			**3.440**	**0.179**
Female	38 (61.3%)	6 (85.7%)		
Male	24 (38.7%)	1 (14.3%)		

**Number of chronic diseases**			**31.794**	**0.000**
	44 (71.0%)	2 (28.6%)		
1	15 (24.2%)	4 (57.1%)		
2	3 (24.8%)	0 (0%)		
3	0 (0%)	1 (14.3%)		
4				

## Discussion

The risk of acquiring, preventing and controlling diseases, especially infectious ones, could depend on individuals' perceived risk.^16^ In this study, the highest proportion of the respondents agreed that they were at risk of being infected with COVID-19 while attending the clinic. This finding may be due to the awareness that people who go to hospitals for other treatment besides COVID-19 are at higher risk of getting infected with the virus than the general public.^27^ Almost one-third of the respondents indicated they would likely become sick with the new COVID-19. These findings were quite comparable with a study in Ecuador, South America, among adult outpatient hypertensives, who reported a higher infection risk.^28^ This may be due to the awareness that those with chronic illnesses are more likely to be infected with COVID-19 than those without chronic illnesses.^29^, ^30^

The older adults, the healthcare workers and those with chronic illnesses were those that the respondents perceived were at the most risk of being infected with COVID-19. Their perception follows what the Centres for Disease Control and Prevention (CDC) reported.^30^ The risk perception of COVID-19 is expected to enhance the uptake of preventive behaviours, including vaccine uptake and acceptance. This study found that more than half of the respondents indicated that using a facemask and regularly washing hands with water and soap could prevent infection with COVID-19. This was also in synchrony with a study conducted in South America in which almost all respondents believed using protective measures and washing hands could prevent infection with COVID-19.^28^ In addition, just above half of the participants agreed that not sitting close to people could prevent COVID-19 and half of the respondents agreed that COVID-19 could be contracted if no preventive measure was taken. This behaviour agrees with a study in Northern Nigeria, which reported that many participants adopted social distancing measures.^33^ A study in Southern Nigeria showed that people who had high risk equally agreed that obeying government lockdown policies, washing their hands regularly or, using hand sanitiser, and using face masks in public places could prevent COVID-19 infection.^34^ This supported our findings that high self-perceived risk in acquiring the disease makes people exhibit better behaviours.

In this study, less than one-fifth of the respondents had ever taken a COVID-19 test. However, only about one-tenth of those tested were positive for COVID-19, and only slightly less than half were hospitalised. This study does not agree with another study in Northwest Ethiopia, which included those with chronic illnesses, who reported that more than one-fifth of the respondents had ever taken a COVID-19 test. It was also reported that almost half of those tested were positive for COVID-19.^23^ The disparity could be that more people had the test in the Ethiopian study than in this study.

Compared with other studies reports among chronic disease patients, this study had a relatively higher COVID-19 vaccine uptake.^23^,^35^ Over two-thirds of the respondents in this study had received at least one dose of the COVID-19 vaccine. The highest proportion of those who received the COVID-19 vaccine had only taken the first dose, followed by those who had up to the booster dose. A study in Northwest Ethiopia reported that less than one-third of the respondents had received at least one dose of COVID-19. About 31.2% had received the vaccine in the first round.^23^ Another study in Ethiopia reported that only 14.5% of the respondents had received the vaccine in the first round and 30.8% in the second round after the rollout of the vaccine.^35^ This study had a higher COVID-19 uptake among those with chronic illnesses because the study period was later than the other studies, and there is a tendency that the study participants had the opportunity to learn more about vaccine safety than those studies conducted earlier.

Out of the respondents (30.5%) who did not receive the COVID-19 vaccine, fear of the unknown was the most typical reason for not taking the vaccine, closely followed by the fear of side effects. This finding was similar to those conducted among cancer patients and those with chronic diseases in different parts of Ethiopia, where those who didn't receive the vaccine were majorly concerned about the safety and fear of side effects of the vaccine.^23^,^35^ Not being sure of vaccine effectiveness and fear of potential adverse effects were two common reasons why those who are chronically ill did not receive the vaccine. This could be because of the uncertainty and inadequate information about the vaccine efficacy and side effects because COVID-19 is a relatively new vaccine.

In this study, the uptake of the COVID-19 vaccine was significantly associated with marital status. Those who were married among the chronically ill were more likely to take the COVID-19 vaccine. However, no other socio-demographic variables like age, sex, or educational status were associated with COVID-19 vaccine uptake. Other studies among those chronically ill reported an association between age, sex and educational status and COVID-19 vaccine uptake.^23^,^35^

Positive risk perception of COVID-19 was associated with COVID-19 vaccine uptake in this study. This is also comparable to another study in Ethiopia, which reported that the chronically ill with a positive attitude towards the COVID-19 vaccine were likelier to take it.^23^ Those individuals with two chronic diseases were seen to be likely to test positive for COVID-19. This supports reports that COVID-19 is commoner and causes severe diseases among those with chronic illnesses.^36^

### Study Limitations

This study is a cross-sectional study. Therefore, it is difficult to establish a causal relationship between the factors explored and the COVID-19 vaccine uptake. The positive result from the COVID-19 test was based on a verbal report from the patient and not the laboratory report.

## Conclusion

About half of the respondents agreed that they were at risk of COVID-19 infection, and vaccine acceptance was notably high in this study. Concerns over unknowns and side effects were primary reasons for vaccine hesitancy among those not yet vaccinated. Marital status and COVID-19 risk perception were the only factors linked to vaccine acceptance.

Stakeholders (Government, health portals, and health workers) should continue enlightening the public, particularly those with chronic illnesses, about the risks of COVID-19 and the need to take precautionary measures such as the COVID-19 vaccine uptake. Also, clear information should be provided to the people about the COVID-19 vaccine and its safety to help increase the COVID-19 vaccine uptake among people, especially the chronically ill who have a higher risk of getting infected and have a severe form of COVID-19.
